# The c.1617del variant of *TMEM260* is identified as the most frequent single gene determinant for Japanese patients with a specific type of congenital heart disease

**DOI:** 10.1038/s10038-024-01225-w

**Published:** 2024-02-26

**Authors:** Tadashi Inoue, Ryuta Takase, Keiko Uchida, Kazuki Kodo, Kenji Suda, Yoriko Watanabe, Koh-Ichiro Yoshiura, Masaya Kunimatsu, Reina Ishizaki, Kenko Azuma, Kei Inai, Jun Muneuchi, Yoshiyuki Furutani, Hiroyuki Akagawa, Hiroyuki Yamagishi

**Affiliations:** 1 Department of Pediatrics, Keio University School of Medicine, Tokyo, Japan; 2 Department of Pediatrics and Child Health, Kurume University School of Medicine, Fukuoka, Japan; 3 Keio University Health Center, Tokyo, Japan; 4 Research Institute of Medical Mass Spectrometry, Kurume University School of Medicine, Fukuoka, Japan; 5 Department of Human Genetics, Division of Advanced Preventive Medical Sciences, Leading Medical Research Core Unit, Nagasaki University Graduate School of Biomedical Sciences, Nagasaki, Japan; 6 Department of Pediatrics, Chiba University Graduate School of Medicine, Chiba, Japan; 7 Institute for Comprehensive Medical Sciences, Tokyo Women’s Medical University, Tokyo, Japan; 8 Department of Pediatric Cardiology and Adult Congenital Cardiology, Tokyo Women’s Medical University, Tokyo, Japan; 9 Department of Pediatrics, Kyushu Hospital, Japan Community Healthcare Organization, Kitakyushu, Japan; 10 Center for Preventive Medicine, Keio University School of Medicine, Tokyo, Japan

**Keywords:** Disease genetics, Congenital heart defects

## Abstract

Although the molecular mechanisms underlying congenital heart disease (CHD) remain poorly understood, recent advances in genetic analysis have facilitated the exploration of causative genes for CHD. We reported that the pathogenic variant c.1617del of *TMEM260*, which encodes a transmembrane protein, is highly associated with CHD, specifically persistent truncus arteriosus (PTA), the most severe cardiac outflow tract (OFT) defect. Using whole-exome sequencing, the c.1617del variant was identified in two siblings with PTA in a Japanese family and in three of the 26 DNAs obtained from Japanese individuals with PTA. The c.1617del of *TMEM260* has been found only in East Asians, especially Japanese and Korean populations, and the frequency of this variant in PTA is estimated to be next to that of the 22q11.2 deletion, the most well-known genetic cause of PTA. Phenotype of patients with c.1617del appears to be predominantly in the heart, although *TMEM260* is responsible for structural heart defects and renal anomalies syndrome (SHDRA). The mouse TMEM260 variant (p.W535Cfs*56), synonymous with the human variant (p.W539Cfs*9), exhibited truncation and downregulation by western blotting, and aggregation by immunocytochemistry. In situ hybridization demonstrated that *Tmem260* is expressed ubiquitously during embryogenesis, including in the development of cardiac OFT implicated in PTA. This expression may be regulated by a ~ 0.8 kb genomic region in intron 3 of *Tmem260* that includes multiple highly conserved binding sites for essential cardiac transcription factors, thus revealing that the c.1617del variant of *TMEM260* is the major single-gene variant responsible for PTA in the Japanese population.

## Introduction

Congenital heart disease (CHD) is caused by defects in cardiovascular development during the first trimester of gestation and represents the most common and life-threatening congenital birth defect among neonates worldwide with an incidence of 8.60–10.25 per 1000 births [1]. Persistent truncus arteriosus (PTA) is one of the most serious CHDs. It is a cardiac outflow tract (OFT) defect characterized by a single arterial trunk arising from the heart, instead of a separate pulmonary artery and aorta that supply systemic, pulmonary, and coronary circulations [2]. Most patients with PTA require surgery in the neonatal period, as the early mortality rate from surgery is 3–20%, and the long-term survival rate at 20 years is approximately 75% [2]. Although the 22q11.2 deletion syndrome is the most well-known genetic factor causing PTA, accounting for 30–50% of cases, and *TBX1* on the 22q11.2 locus is the determinant gene [3–5], the molecular mechanisms by which PTA occurs during embryogenesis are not fully understood. To date, several causative genes have been reported, including *TBX1*, *NKX2-5*, *NKX2-6*, *GATA4*, and *GATA6* [6] in syndromic PTA or, more rarely, in isolated PTA.

The *TMEM260* gene, which is located on chromosome 14 and encodes 707 amino acids, encodes a mannosyl-transferase [7] whose biallelic variant is associated with structural heart defects and renal anomaly syndrome (heart, kidney, and nerve symptoms: SHDRA syndrome [OMIM# 617478]) [8]. Recently, *TMEM260* variants were reported in Japanese patients with syndromic CHD, particularly PTA [9]. The impact of *TMEM260* variants in the Japanese population and how TMEM260 is associated with cardiac development and PTA genesis remain to be studied.

In this study, we identified the c.1617del variant of *TMEM260* in a family with PTA and in multiple DNAs from patients with PTA in our genome bank, using a cell line obtained and established from numerous CHD patients. Through combinatorial analysis using public databases, genotype-phenotype correlation, and expressional and functional analyses of *TMEM260* and its variants, our study revealed that the c.1617del variant of *TMEM260* is a major genetic cause of PTA in the Japanese population.

## Materials and methods

### Participants and genetic analyses

We performed whole-exome sequencing (WES) including two probands to one family and their healthy parents and sister who were diagnosed and hospitalized in Kurume University Hospital as part of the “Initiative on Rare and Undiagnosed Diseases in Pediatrics (IRUD-P)” project in Japan.

Next, from the genome bank with cell lines in Japan [10, 11], 104 cases with OFT defects (26 with PTA and 78 with pulmonary atresia with ventricular septal defect [PA-VSD]) were analyzed after exclusion of genetic disorders including 22q11.2 deletion syndrome (Supplementary Fig. S1). Genomic DNA (gDNA) was extracted, and genetic analysis was performed as described previously [10, 11] (details are in the Supplementary Materials).

As a control, our WES datasets from 143 individuals without congenital disease and 95 non-congenital disease controls were outpatients of Tokyo Women’s Medical University Hospital and its neighboring affiliated hospitals [12].

Candidate variants were verified by Sanger sequencing using gDNA from peripheral blood leukocytes or the lymphoblastoid cell line (F6-II-2 in Fig. 2) with the primers listed in Supplementary Table S1.

### Cloning, mutagenesis, and plasmid construction of the full-length mouse *Tmem260*

Mouse *Tmem260* complementary DNA (cDNA) was cloned from the cDNA library generated from embryonic day, E 10.5 by TA cloning with the primers shown in Supplementary Table S1 using pGEM®-T easy vector (Promega, Madison, WI, USA). Site-directed mutagenesis was performed using the KOD Plus Mutagenesis Kit (Toyobo, Osaka, Japan). Polymerase chain reaction products tagged at both ends with restriction enzyme recognition sites (primers are shown in Supplementary Table S1) were subcloned into the pCMV-Tag3 expression vector (Agilent Technologies, Santa Clara, CA, USA).

### Western blotting

Proteins were prepared from HEK293T cells transfected with expression vectors using Lipofectamine 2000. Cells were lysed on ice in cell lysis buffer (Cell Signaling Technology, Danvers, MA, USA) containing 1 mM Phenylmethylsulfonyl fluoride for 10 min. The lysates were immediately resolved to TGX Gel 4–20% (Bio-Rad, Hercules, CA, USA) by sodium dodecyl sulfate-polyacrylamide gel electrophoresis and transferred to polyvinylidene fluoride membranes for 7 min at 2.5 A, using the Trans-Blot® Turbo^TM^ Transfer System (Bio-Rad). c-Myc-tagged TMEM260 proteins were detected by western blotting using a monoclonal anti-c-Myc antibody (BioLegend, San Diego, CA, USA), goat anti-mouse IgG-HRP-conjugated antibody (Santa Cruz Biotechnology, Santa Cruz, CA, USA), and SuperSignal West Femto Maximum Sensitivity Substrate (Thermo Fisher Scientific, Waltham, MA, USA).

### Immunocytochemistry

Twenty-four to forty-eight hours after transient transfection with Lipofectamine 2000, HEK293T cells grown on coverslips were fixed with 2% formaldehyde/medium, permeabilized with 0.1% TritonX-100/PBS, incubated with monoclonal anti-c-Myc antibody (BioLegend), polyclonal anti-Calreticulin antibody (Thermo Fisher Scientific) and DAPI (Wako, Osaka, Japan), and detected using anti-mouse Alexa Fluor 488 or anti-rabbit Alexa Fluor 546 antibodies (Thermo Fisher Scientific). A laser confocal microscope (TCSSP5, Leica Microsystems, Wetzlar, Germany) was used to capture images.

### In situ hybridization

Whole-mount in situ hybridization with digoxigenin-labeled riboprobe for *Tmem260* was performed on whole embryos within the E8.5–11.5 development range, as well as on hearts isolated from E10.5 to E11.5 ICR mice. This procedure followed the methodology as described previously, with minor modifications [13]. Riboprobes were synthesized from an 827-bp fragment (NM_172600.4 c.510-1336) amplified from full-length *Tmem260* cDNA. Images were obtained under a microscope, MZ9.5 (Leica Microsystems, Wetzlar, Germany) and captured using a color CCD camera, DP27 (Olympus, Tokyo, Japan).

### Enhancer analysis using transgenic mouse embryos

A specific region of the mouse *Tmem260* intron 3 (NM_172600.4 c.332+4469 to +5276) was subcloned into the hsp68-lacZ reporter construct for the mouse *Tmem260* enhancer-lacZ plasmid. DNA fragments were microinjected to generate transgenic mice. Transgene expression was confirmed by PCR analysis of lacZ using the yolk sac DNA. Harvested embryos (E10.5) were incubated in a staining solution containing X-gal after fixation, as described previously, with minor modifications [13].

## Results

### c.1617del variant of *TMEM260* was identified as being responsible for PTA in a Japanese family

**Fig. 1:**
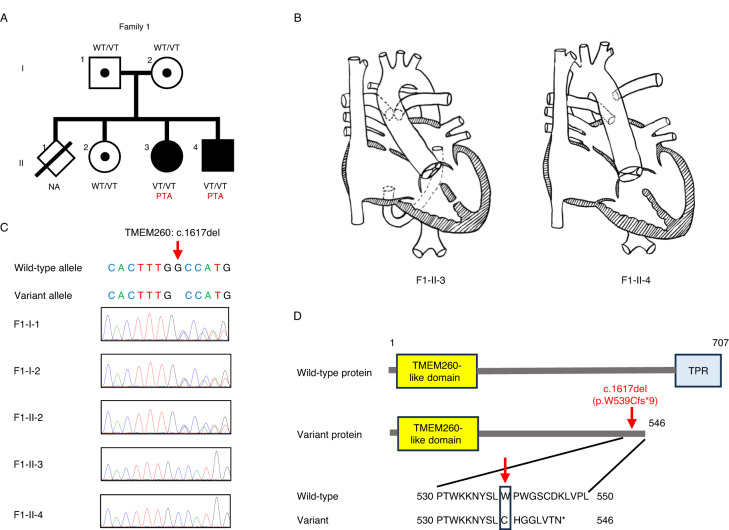
The first family affected by the c.1617del variant of *TMEM260*. **A** Pedigree chart of the family showing members with confirmed variants of persistent truncus arteriosus. Affected individuals are denoted by filled symbols. **B** Cardiac phenotype of two probands. F1-II-3 Persistent truncus arteriosus (type II in Collett and Edwards classification) with quadricuspid truncal valve, two ventricular septal defects, aberrant right subclavian artery, and collateral vessel from the left atrium to the right atrium. F1-II-4 Persistent truncus arteriosus (type III in Collett and Edwards classification) with tricuspid truncal valve, ventricular septal defect, and right aortic arch. **C** Sequence chromatograms of the *TMEM260* gene (NM_017799.4) are shown. The c.1617del variant in the *TMEM260* gene (red arrow) was observed to be heterozygous in F1-I-1, F1-I-2, and F1-II-2 and homozygous in F1-II-3 and F1-II-4. **D** The structures of the human *TMEM260* genes, and the positions of the variant (red arrow) are shown. Changes in amino acid are shown in the rectangle. TPR, tetratricopeptide

The Japanese family of interest included two patients with PTA and two siblings of healthy, nonconsanguineous parents (Fig. 1A). The first patient (Fig. 1A; F1-II-3) was a 3-year-old girl born at 39 weeks of gestation with a birth weight of 2.618 kg. She was diagnosed with PTA type II by the Collett and Edwards classification, with an aberrant right subclavian artery, ventricular septal defects (VSDs) at the subaortic and muscular regions, and collateral vessels from the left atrium to the right atrium (Fig. 1B). The patient had no dysmorphic features, and the structure and function of her kidneys were normal. She had left-sided hearing loss but no developmental delay, and magnetic resonance imaging of her brain at the age of 4 years was normal. The second patient (Fig. 1A; F1-II-4), the younger brother of the first, was a 1-year-old boy born at 39 weeks of gestation with a birth weight of 2.838 kg. He was diagnosed with PTA type II according to the Collett and Edwards classification, with a subaortic VSD and right aortic arch (RAA) (Fig. 1B). He did not have any dysmorphic features or structural or functional abnormalities of his kidneys, and development with brain magnetic resonance imaging at the age of one year was normal. One of their siblings (Fig. 1A: F1-II-1) died as a neonate with no information about his/her phenotype, while the other (Fig. 1A: F1-II-2) was a healthy female.

WES analysis was performed to identify the genetic cause of PTA in this family. Homozygous variants were detected in exon 13 of *TMEM260* (NM_017799.4 c.1617del) in the first and second patients (Fig. 1A; F1-II-3, F1-II-4). Subsequently, they were validated to harbor this c.1617del variant in the *TMEM260* homozygously, while the unaffected parents and child were heterozygous as determined by Sanger sequencing (Fig. 1C). The MAF for the c.1617del variant in the in-house control group was 0.0021 (allele 1/476), 0.000021 in the gnomAD database [14], and 0.0036 and 0.0029 in the Japanese population [15, 16], indicating that this is a rare variant. The minor allele frequency of the c.1617del variant of *TMEM260* is summarized in Supplementary Table S2. This frameshift variant leads to a change from tryptophan to cysteine in amino acid 539 and the creation of a stop codon after the ninth amino acid, resulting in the variant TMEM260 protein, which shortens the length from 707 to 546 amino acids (p.W539Cfs*9). With this change, the TMEM260 variant protein loses its functional domain of a substrate recognition region called the tetratricopeptide repeat (TPR) (AA 614–696) (Fig. 1D). In addition, the c.1617del variant was predicted to be disease causing by MutationTaster [17], with a CADD score of 33 (highly deleterious) [18]. It has also been reported that *TMEM260* variants caused the SHDRA syndrome, which is strongly associated with PTA [7–9, 19, 20]. Taken together, these results suggest that the homozygous variant c.1617del of *TMEM260* causes PTA in this family in an autosomal recessive manner.

### c.1617del variant of *TMEM260* may be a major genetic cause for PTA in Japanese patients

**Fig. 2:**
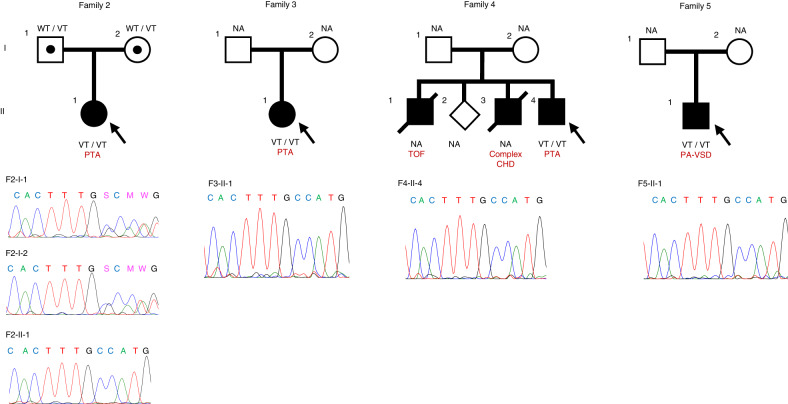
Four pedigrees affected by the c.1617del variant of *TMEM260* and their sequence chromatograms. Participants with and without CHD are shown as filled and unfilled symbols, respectively. CHD congenital heart disease, NA not analyzed, PA-VSD pulmonary atresia with ventricular septal defect, PTA persistent truncus arteriosus, TOF Tetralogy of Fallot, VT variant type, WT wild-type

To elucidate the impact of the c.1617del variant of *TMEM260*, we examined *TMEM260* in twenty-six consecutive Japanese individuals with PTA using our genome bank with cell lines from patients with numerous CHDs [21]. Next-generation sequencing analysis revealed the homozygous c.1617del variant of *TMEM260* in three (12%) of the 26 patients with PTA. Pedigree charts of the patients and Sanger sequencing traces of the variant used for validation are shown in Fig. 2. We also searched for the same variant in the patient’s parents, whose gDNA was available in our genome bank, and detected that the variant was heterozygous in Family 2. Furthermore, we analyzed DNA from 78 consecutive Japanese patients with PA-VSD, which is categorized into the same developmental spectrum of CHD with PTA [22], using the same genome bank, and found the homozygous c.1617del variant of *TMEM260* in one patient (Fig. 2, Family 5). In the 104 cases that we examined, the 22q11.2 microdeletion was previously excluded, and the absence of any pathogenic variants in *GATA6*, *GATA4*, *NKX2.5*, *MEF2C* and *ISL1* genes was confirmed by exon-based sequencing [11]. Intriguingly, in all five patients with PTA and one patient with PA-VSD who were associated with the c.1617del variant, neither neurological disorders nor structural abnormalities in the kidney were documented, as in the two affected children in family 1. Four of the five patients with PTA and one patient with PA-VSD also had aortic abnormalities such as RAA, interruption of the aortic arch, and aberrant right subclavian artery. The phenotypes are summarized in Table 1.

**Table 1:** List of the phenotype information for 6 newly reported patients with *TMEM260* c.1617del variant

Family	Individual ID	Gender	Age	Race	Consanguinity	Genotype	Cardiac phenotype		Other SHDRA-related phenotypes	
							OFT	Aorta	Neurological	Renal
Family 1	F1-II-3	F	17 months	Japanese	–	homo	PTA	ARSCA	–	–
	F1-II-4	M	15 days	Japanese	–	homo	PTA	RAA	–	–
Family 2	F2-II-1	F	6 years	Japanese	–	homo	PTA	RAA	–	–
Family 3	F3-II-1	F	22 years	Japanese	–	homo	PTA	–	–	–
Family 4	F4-II-4	M	2 days	Japanese	–	homo	PTA	RAA	NA	–
Family 5	F5-II-1	M	7 years	Japanese	–	homo	PA-VSD with MAPCAs	RAA	–	–

*ARSCA* aberrant right subclavian artery, *MAPCAs* major aortopulmonary collateral arteries, *NA* not analyzed, *OFT* outflow tract, *PA-VSD* pulmonary atresia with ventricle septal defect, *PTA* persistent truncus arteriosus, *RAA* right aortic arch, *-* none

### Significance of the c.1617del variant in the *TMEM260* gene

**Fig. 3:**
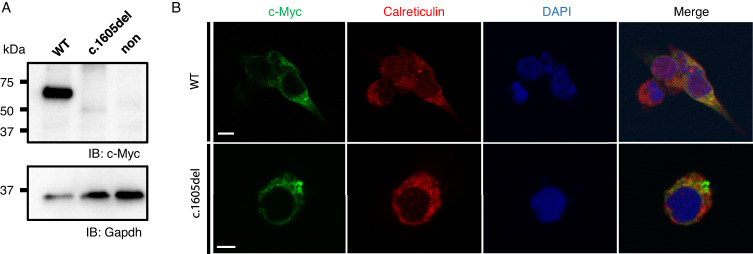
Mouse *Tmem260* c.1605del variant shows protein instability and aggregation. **A** Western-blot analysis of HEK293T cells transfected with c-Myc tagged *Tmem260* wild-type or variant gene. **B** Immunofluorescent staining of HEK293T cells transfected with c-Myc tagged *Tmem260* wild-type or variant, by anti-c-Myc and anti-Calreticulin antibodies. Scale bar was 5 µm. IB immunoblot, WT wild-type

To verify the pathogenicity of the c.1617del variant on *TMEM260* function, quantitative PCR showed that *TMEM260* mRNA expression was significantly decreased in cases with homozygous c.1617del variant compared to the in-house control cases (Welch’s test, *p* = 0.008, Supplementary Fig. S2). Next, we assessed the expression of variant proteins in vitro. Mouse *Tmem260* shares a high degree of homology to its human counterpart, and the c.1605del (p.W535Cfs*56) variant of mouse *Tmem260* is synonymous with the c.1617del variant found in human *TMEM260*. The mouse TMEM260 p.W535Cfs*56 variant lost its TPR functional domain, reminiscent of the human TMEM260 p.W539Cfs*9 variant. Wild-type and variant mouse *Tmem260* cDNAs were subcloned into a c-Myc-tagged expression vector and used for subsequent experiments. Western blot analyses showed the molecular weight of the variant TMEM260 protein was about 50 kDa, smaller than the wild-type TMEM260, whose molecular weight was about 60 kDa. In addition, the amount of the variant protein transfected into HEK293T cells was less than that of the wild-type protein. One possible interpretation is that insoluble aggregates in the sample buffer for western blotting, discarded as remnants, or decreased stability of the variant protein compared to the wild-type protein (Fig. 3A).

Next, immunocytochemistry was performed to assess the intracellular expression of TMEM260. First, we confirmed that the c-Myc-tagged wild-type TMEM260 protein was localized properly in endoplasmic reticulum as previously reported [7], demonstrated by co-immunostaining with anti-Calreticulin antibody (Fig. 3B). However, the TMEM260 p.W535Cfs*56 variant-expressing cells were found very few and the variant protein was aggregated in the cytoplasm of HEK293T cells (Fig. 3B), suggesting an abnormal function of this variant protein.

### Expression pattern of *Tmem260* during embryogenesis

**Fig. 4:**
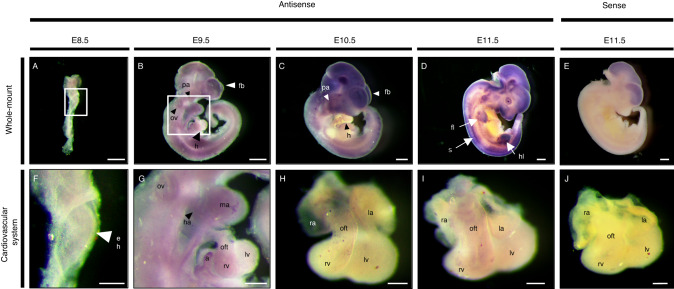
*Tmem260* is expressed in murine embryos including in the heart. **A**–**E** Whole-mount in situ hybridization using mouse embryos from E8.5 to E11.5 developmental stages, and the scale bar is 500 µm. **F**, **G** Enlarged views of the heart region enclosed by the white square in (**A**, **B**), respectively. **H**–**J** Whole-mount in situ hybridization using isolated embryonic hearts. The scale bars in (**F**–**J**) are 200 µm. The panels of (**E**, **J**) show in situ hybridization with sense probe. a atrium, eh embryonic heart, fb frontal brain, fl forelimb, h heart, ha hyoid arch, hl hind limb, la left atrium, lv left ventricle, ma mandibular arch, oft outflow tract, ov otic vesicle, pa pharyngeal arch, ra right atrium, rv right ventricle, s somite

If the abnormality of TMEM260 expression and function is associated with PTA, TMEM260 should be expressed in the embryonic heart from E8.5 to 11.5, an important period for cardiac OFT development. Therefore, we performed in situ hybridization with antisense and sense riboprobes for *Tmem260* in murine embryos to confirm the expression of *Tmem260*. *Tmem260* was ubiquitously expressed with some variation among tissues and organs throughout development (Fig. 4). At E8.5, the expression level of *Tmem260* was very low but was relatively enhanced in the embryonic heart (Fig. 4A, F). At E9.5, *Tmem260* expression becomes clearer, and the expression signals were enhanced in the mandibular arches, hyoid arches, atria, and right ventricle of the OFT (Fig. 4B, G). In E10.5 and E11.5, *Tmem260* expression was detectable in the heart, with dominant expression in other tissues and organs, including the head, limb buds, and somites (Fig. 4C, D). The expression of *Tmem260* was also confirmed in the OFT by in situ hybridization performed on hearts isolated from E10.5 or E11.5 murine embryos (Fig. 4H, I). In situ hybridization using the sense RNA probe on whole-mount embryos and isolated hearts at E11.5 used as a negative control (Fig. 4E, J).

### Putative transcriptional regulation of *Tmem260* expression during heart development

**Fig. 5:**
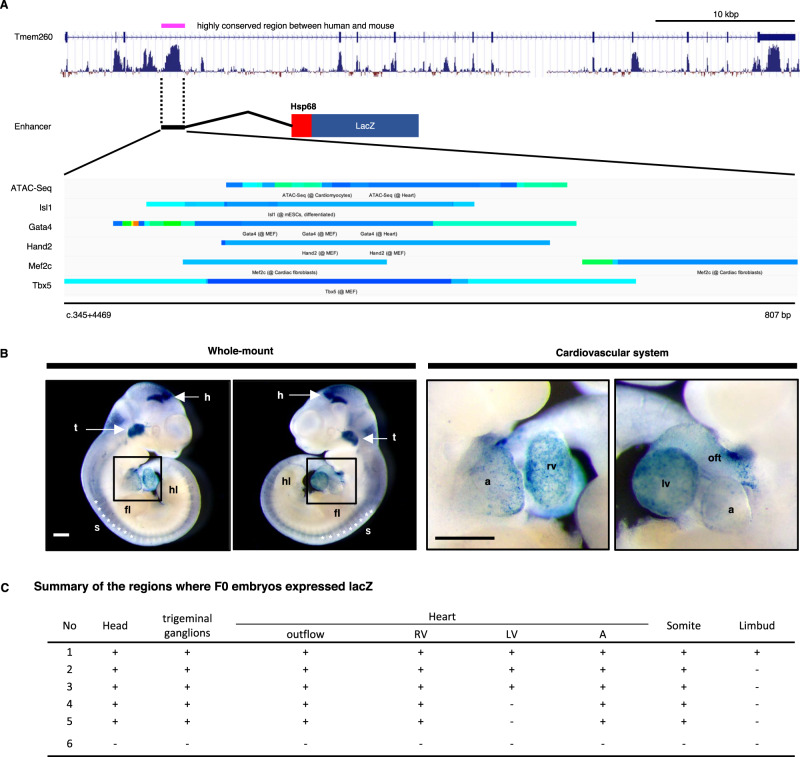
The region in *Tmem260* intron 3 is a putative cardiac enhancer. **A** In the upper panel, the structure of the mouse *Tmem260* locus and flanking region with the conservation score among 100 vertebrates. A schema of a mouse 807 bp *Tmem260* enhancer-lacZ reporter construct in the middle. The 807 bp region has previously-reported binding sites for multiple transcription factors with open chromatin identified using the ChIP-Atlas database. **B** Lateral views of representative embryos obtained with this construct are shown in the two left panels. The two pictures on the right show a magnified view of the heart. Scale bars are 500 µm. **C** Summary of the regions where lacZ expression was detected in F0 embryos. a atrium, fl forelimb, h head, hl hind limb, lv left ventricle, oft outflow tract, rv right ventricle, s somite, t trigeminal ganglia

To delineate the molecular mechanisms that regulate the expression of *Tmem260* during heart development, we analyzed *Tmem260* genomic sequences and searched for cis-regulatory elements that may control *Tmem260* transcription using the online tool Chip-Atlas (https://chip-atlas.org/) [23]. Interestingly, we found that an 807-bp region of intron 3 in the *Tmem260* locus of the mouse genome (NM_172600.4 c.332+4469 to +5276) and the corresponding human genome locus (NM_017799.4 c.345+4564 to +5371) contained an open chromatin region, as determined by transposase-accessible chromatin sequencing. This region contains binding sites for multiple transcription factors, including *Isl1*, *Tbx5*, *Gata4*, *Hand2*, and *Mef2c*, which are essential for heart development (Fig. 5A) as determined by chromatin immunoprecipitation sequencing [23]. The homology of the regions was estimated to be as high as 94.5% (763 bp out of 807 bp were identical) among the 100 vertebrate species using the UCSC genome browser (https://genome.ucsc.edu/). To determine whether this 807-bp region contains regulatory elements responsible for *Tmem260* expression in the developing heart, we tested its ability to direct lacZ expression under a heterologous promoter (hsp68) using a transgenic mouse system. F0 transgenic mouse embryos harboring the 807-bp transgene of the *Tmem260* intron 3 region showed X-gal staining in the head, trigeminal ganglia, somite, and heart, especially in the OFT, right ventricle, and atria at E10.5 (Fig. 5B, C). These results suggest that this 807-bp DNA sequence may at least in part act as a weak enhancer of *Tmem260*, putatively via regulation by essential cardiac transcription factors during heart development.

## Discussion

In the present study, the c.1617del variant of *TMEM260*, a rare variant specific to East Asians, was identified in several Japanese patients with PTA. The c.1617del variant induces protein truncation, resulting in the deletion of the C-terminal functional domain (TRP) of TMEM260, which may affect the protein function of TMEM260, probably by altering protein stability and resulting in protein aggregation in the cell. Our study using mouse embryos showed that *Tmem260* is ubiquitously expressed during embryogenesis, including in the developing heart, putatively through the regulation of essential cardiac transcription factors. These results strongly suggest that the c.1617del mutation in *TMEM260* is a pathogenic variant of PTA in the Japanese population.

The biallelic variant of *TMEM260* has been shown to be responsible for SHDRA syndrome, which is highly associated with PTA [8]; thus far, 17 cases from 11 families have been reported (Supplementary Table 3). Among them, only one East Asian patient harbored the c.1617del variant as a compound heterozygote associated with PTA [9]. Of note, all our patients with the c.1617del variant showed PTA or PA-VSD (in one case), but no other phenotype of SHDRA syndrome, whereas approximately half of the SHDRA syndrome patients showed neurological or renal abnormalities in previous reports [7–9, 19, 20]. Moreover, more cases of aortic arch abnormalities were observed in our study than in previous reports (87.5% [7/8cases] vs. 29.4% [5/17cases]). These results suggest that the c.1617del variant of *TMEM260* may be more specifically associated with the cardiovascular phenotype than other phenotypes of SHDRA, making the homozygous c.1617del variant of *TMEM260*, in particular, a genetic cause for isolated PTA or isolated PA-VSD in a few cases, in an autosomal recessive manner. Because the mRNA level of the cell lines from the homozygous c.1617del variant cases appeared to be down, but almost half was still expressed according to our quantitative PCR analysis (Supplementary Fig. S2), this relatively high residual mRNA expression might contribute to normal neurological and renal development, although the mutant proteins lost the localization. On the other hand, the previous studies have included individuals with various backgrounds different from our patients that may influence the phenotypic difference. To the best of our knowledge, this is the first report showing an autosomal recessive gene variant causing a specific type of CHD in Japan, although there has been only one report showing that the homozygous *GDF1* c.1091 T > C variant, a rare inherited variant, causes several types of CHD in Ashkenazim [24].

According to the gnomAD [14] and Allele Frequency Aggregator (www.ncbi.nlm.nih.gov/snp/docs/gsr/alfa/) international databases, as well as the Japanese populations, the c.1617del variant of *TMEM260* is a rare variant that has been reported only in a specific population of East Asians, especially Japanese and Koreans. In this study, we identified this homozygous variant in three of 26 DNAs from consecutive patients with PTA, suggesting that the c.1617del variant of *TMEM260* might account for approximately 12% of PTA cases, although its precise frequency remains unknown. This frequency could make this variant the second known major genetic cause of PTA after the chromosomal microdeletion of 22q11.2, which account for 30–50% [3], 34–41% [4], and 33% [5] of PTA cases in Italy, USA, and Japan, respectively. The c.1617del variant of *TMEM260* is the most common single-gene variant associated with PTA in Japan. Taken together, we speculate that the c.1617del variant of *TMEM260* might be a founder variant for PTA in Japan or East Asia because it is responsible for rare genetic disorders, usually resulting from recessive alleles, with a relatively high frequency in specific populations. However, more detailed analyses, such as haplotype analysis, are needed to confirm this [25].

In this study, two patients (F6-II-2 and F7-II-2 in Supplementary Fig. S3 and Supplementary Table S4) with PTA harbored only one allele of the c.1617del variant instead of the biallelic variant. We are not able to explain how these patients developed PTA. If we conduct WES or copy number variant analysis using NGS data in a future study, it may provide new insights of rare variants in other genes related to cardiac OFT development, such as *TBX1* or *NKX2-6*, or exonic deletions/duplications in *TMEM260*. One explanation of why the c.1617del variant might result in susceptibility to PTA even if it is monoallelic, is because the allele frequency of this variant is higher in the PTA population (MAF 0.031: 2/64 allele) than in the healthy population (MAF 0.0021:1/476 allele). Other possible reasons may be as follows: (1) another allele of *TMEM260* may have some alteration that cannot be detected by exon sequencing, such as variations in the untranslated region or deep-intron region in *TMEM260*; (2) variants in genes other than *TMEM260* cause PTA in concert with the monoallelic c.1617del variant; and (3) non-genetic factors such as environmental factors play a role in concert with the monoallelic c.1617del variant.

The mechanism underlying the *TMEM260* variant (c.1617del, p.W539Cfs*9) that specifically results in PTA remains unclear. Our data suggest that the C-terminal region from amino acid 539 onwards, including the TPR domain, may be critical for the development of cardiac OFT, and truncation of this region may be associated with PTA or rarely with PA-VSD. Our results also suggest that the reduced size, stability, and aggregation of the TMEM260 protein by p.W539Cfs*9 during heart development might be responsible for PTA. In this study, we showed that the expression of *Tmem260* in mouse embryonic cardiac OFT may be regulated at least in part by the enhancer region in intron 3 of *Tmem260* involving many binding sites for transcription factors essential for cardiovascular development. In particular, the expression of *Tmem260* in the OFT was consistent with the expression of *Plxna2*, an important molecule involved in OFT development [26]. TMEM260 has an enzyme-active domain called the protein O-mannosyl-transferase TMEM260-like domain (amino acids 52–211) in the N-terminal transmembrane region and is biologically active by transferring mannose to the PLEXIN family [7]. It would be interesting to investigate whether the altered function of *TMEM260* may result in PTA due to a failure in the activation of molecules essential for cardiac OFT development, such as the PLEXIN family, in the future, by creating an animal model.

In conclusion, the c.1617del variant of *TMEM260* that might be a “founder variant” specific for the cardiac phenotype, is revealed to be the most frequent single gene variant responsible for PTA in Japanese populations. A genetic test for variants of *TMEM260*, including the c.1617del variant, is recommended for Japanese patients with PTA, especially in cases excluding 22q11.2 syndrome, in order to provide better genetic counseling. Simultaneously, a study group at Tohoku University reported the same c.1617del variant of *TMEM260* as a major genetic cause of PTA, resulting in the same conclusion as that of our study (Prof. A. Kikuchi at Tohoku University and H. Yamagishi, personal communication). Therefore, we propose to name this c.1617del variant as “Keio-Tohoku variant of *TMEM260*”, considering its importance in the Japanese population.

### Supplementary information

Supplementary Information

Figure S1

Figure S2

Figure S3

Table S1

Table S2

Table S3

Table S4

## References

[1] Liu Y, Chen S, Zü L, Black GC, Choy M-K, Li N (2019). Global birth prevalence of congenital heart defects 1970-2017: updated systematic review and meta-analysis of 260 studies. Int J Epidemiol.

[2] Naimo PS, Konstantinov IE (2021). Surgery for truncus arteriosus: contemporary practice. Ann Thorac Surg.

[3] Unolt M, Versacci P, Anaclerio S, Lambiase C, Calcagni G, Trezzi M (2018). Congenital heart diseases and cardiovascular abnormalities in 22q11.2 deletion syndrome: From well-established knowledge to new frontiers. Am J Med Genet A.

[4] Goldmuntz E (2020). 22q11.2 deletion syndrome and congenital heart disease. Am J Med Genet C Semin Med Genet.

[5] Momma K, Ando M, Matsuoka R (1997). Truncus arteriosus communis associated with chromosome 22q11 deletion. J Am Coll Cardiol.

[6] Yamagishi H. Human Genetics of Truncus Arteriosus. In: Silke Rickert-Sperling, Robert G. Kelly, David J. Driscoll, editors. Congenital Heart Diseases: The Broken Heart. 1st ed. Springer Vienna Ltd; 2016. p. 559–67.

[7] Larsen ISB, Povolo L, Zhou L, Tian W, Mygind KJ, Hintze J (2023). The SHDRA syndrome-associated gene TMEM260 encodes a protein-specific O-mannosyltransferase. Proc Natl Acad Sci.

[8] Ta-Shma A, Khan TN, Vivante A, Willer JR, Matak P, Jalas C (2017). Mutations in TMEM260 cause a pediatric neurodevelopmental, cardiac, and renal syndrome. Am J Hum Genet.

[9] Kuroda Y, Saito Y, Enomoto Y, Naruto T, Mitsui J, Kurosawa K (2023). PHACES-like syndrome with TMEM260 compound heterozygous variants. Am J Med Genet A.

[10] Yoshida MC, Satoh H, Sasaki M, Semba K, Yamamoto T, Toyoshima K (1986). Regional location of a novel yes-related proto-oncogene, syn, on human chromosome 6 at band q21. Jpn J Cancer Res.

[11] Kodo K, Nishizawa T, Furutani M, Arai S, Ishihara K, Oda M (2012). Genetic analysis of essential cardiac transcription factors in 256 patients with non-syndromic congenital heart defects. Circul J.

[12] Maegawa T, Akagawa H, Onda H, Kasuya H (2022). Whole-exome sequencing in a Japanese multiplex family identifies new susceptibility genes for intracranial aneurysms. PLoS One.

[13] Yamagishi H, Olson EN, Srivastava D (2000). The basic helix-loop-helix transcription factor, dHAND, is required for vascular development. J Clin Investig.

[14] Karczewski KJ, Francioli LC, Tiao G, Cummings BB, Alföldi J, Wang Q (2020). The mutational constraint spectrum quantified from variation in 141,456 humans, Genome Aggregation Database Consortium. Nature.

[15] Higasa K, Miyake N, Yoshimura J, Okamura K, Niihori T, Saitsu H (2016). Human genetic variation database, a reference database of genetic variations in the Japanese population. J Hum Genet.

[16] Tadaka S, Hishinuma E, Komaki S, Motoike IN, Kawashima J, Saigusa D (2021). jMorp updates in 2020: Large enhancement of multi-omics data resources on the general Japanese population. Nucleic Acids Res.

[17] Steinhaus R, Proft S, Schuelke M, Cooper DN, Schwarz JM, Seelow D (2021). MutationTaster2021. Nucleic Acids Res.

[18] Rentzsch P, Schubach M, Shendure J, Kircher M (2021). CADD-Splice—improving genome-wide variant effect prediction using deep learning-derived splice scores. Genome Med.

[19] Pagnamenta AT, Jackson A, Perveen R, Beaman G, Petts G, Gupta A (2022). Biallelic TMEM260 variants cause truncus arteriosus, with or without renal defects. Clin Genet.

[20] Peng M, Jing S, Duan S, Lu G, Zhou K, Hua Y (2023). A novel homozygous variant of TMEM260 induced cardiac malformation and neurodevelopmental abnormality: case report and literature review. Front Med (Lausanne).

[21] Hirayama‐Yamada K, Kamisago M, Akimoto K, Aotsuka H, Nakamura Y, Tomita H (2005). Phenotypes with GATA4 or NKX2.5 mutations in familial atrial septal defect. Am J Med Genet A.

[22] Kirby ML (2008). Pulmonary atresia or persistent truncus arteriosus: is it important to make the distinction and how so we do it?. Circ Res.

[23] Oki S, Ohta T, Shioi G, Hatanaka H, Ogasawara O, Okuda Y (2018). ChIP‐Atlas: a data‐mining suite powered by full integration of public ChIP‐seq data. EMBO Rep..

[24] Jin SC, Homsy J, Zaidi S, Lu Q, Morton S, Depalma SR (2017). Contribution of rare inherited and de novo variants in 2,871 congenital heart disease probands. Nat Genet.

[25] Jain A, Sharma D, Bajaj A, Gupta V, Scaria V (2021). Founder variants and population genomes—Toward precision medicine. Adv Genet.

[26] Brown CB, Feiner L, Lu M-M, Li J, Ma X, Webber AL (2001). PlexinA2 and semaphorin signaling during cardiac neural crest development. Development.

