# Supplementary material for: The c.1617del variant of TMEM260 is identified as the most frequent single gene determinant for Japanese patients with a specific type of congenital heart disease
Source: J Hum Genet. 2024 Feb 26;69(5):215–22. doi: 10.1038/s10038-024-01225-w (PMC11043032; doi:10.1038/s10038-024-01225-w)
Supplement: Supplementary file 1 — Supplementary Information [file 10038_2024_1225_MOESM1_ESM.docx]

**Supplementary information**

**Case reports of the patients in Families 2 to 7**

F2-II-1, a female, persistent truncus arteriosus (PTA) (type II in the Collett and Edwards classification), right aortic arch (RAA), and coronary arteriovenous fistula were detected. She was homozygous for the c.1617del variant and her healthy parents were heterozygous for the same variant in the *TMEM260* gene, as expected. F3-II-1, a female, was diagnosed with PTA (type I according to the Collett and Edwards classification). She presented with left pulmonary cystic bronchiectasis, chronic thyroiditis, and a liver cyst. She was homozygous for the c.1617del variant and her parents were not analyzed. Patient F4-II-4, a male, was diagnosed with PTA (type I in the Collett and Edwards classification), severe truncal valve stenosis, RAA, and high take-off of the right coronary artery. At the time of diagnosis, he was in shock with a pH of 7.27 and base excess of-16.7. He had severe truncal valve stenosis (right ventricular pressure, 84/18 mmHg; aortic pressure, 43/25 mmHg), and after emergency balloon truncal valve angioplasty and peritoneal dialysis. The patient underwent intracardiac repair but died perioperatively. The patient was homozygous for this variant. His parents were intact, but his brother died of Tetralogy of Fallot when he was 10 months old, and another brother died of complex congenital heart disease 5 days later. None of his family members were analyzed. Patient F5-II-1, a male, was diagnosed with PA-VSD, major aortopulmonary collateral arteries, RAA, and a partial anomalous pulmonary vein connection. The patient presented with scoliosis and sudden idiopathic deafness. He was homozygous for the c.1617del variant and his parents were not analyzed. In F6-II-2, a female PTA (type II in the Collett and Edwards classification) was detected. She had low-set ears, acute right low-tone sensorineural hearing loss, and scoliosis. The patient was heterozygous for the c.1617del variant, her healthy father was heterozygous for the same *TMEM260* variant, and her healthy mother had no variants in the *TMEM260* gene by exon base analysis. In F7-II-2, a male, PTA (type I in the Collett and Edwards classification), Type B interruption of the aorta (IAA), aberrant left subclavian artery (ALSCA), and left pulmonary vein connected to the hepatic vein were detected. He experienced respiratory failure and shock. Due to his condition and partial anomalous pulmonary vein connection, he was inoperable and died during the neonatal period. The patient was heterozygous for the c.1617del variant, and her healthy parents and sister were not subjected to genetic analyses. All eight patients had outflow defects. Seven of the eight patients had no neurological or renal structural abnormalities, other than F4-II-4. F4-II-4’s renal failure was thought to be pre-renal due to cardiogenic shock caused by severe outflow tract stenosis and it improved after treatment of the stenosis.

**Details of genetic analyses**

Sets of gDNA from the members of Family 1 were subjected to whole-exome sequencing (WES) according to the IRUD-P project using the Agilent SureSelect Exome Target Enrichment System v5 (Agilent Technologies, Santa Clara, CA, USA), followed by paired-end sequencing on Hiseq2500 sequencers (Illumina, San Diego, CA, USA). To identify *de novo* variants, heterozygous variants in the patient and homozygous reference allele variants in the parents were filtered. The Parabricks v3.7.0-1 germline pipeline best practice based on BWA version 0.7.15 and GATK v4.2.0.0 was utilized. Single nucleotide variations and insertions/deletions were annotated using the ANNOVAR software [1]. Substitutions that met the following criteria were selected as “deleterious”: (1) variants: lead stop gain, stop loss, nonsynonymous, or splice site variants according to GENCODE basic version 19 downloaded from the UCSC genome browser; (2) alternative allele frequencies at variant loci ≤0.5% in the following databases: gnomAD2.1.1 in Genome Aggregation Database (<https://gnomad.broadinstitute.org/>) and SNV allele frequency 54KJPN-ANV/INDEL dataset in ToMMo (<https://jmorp.megabank.tohoku.ac.jp/>). In addition, (3) assuming the inheritance patterns, *de novo*, homozygous, and compound heterozygous variants were identified.

Ion Torrent^TM^ semiconductor sequencing, by an Ion AmpliSeq^TM^ Custom Panel using an Ion AmpliSeq^TM^ Designer software (Thermo Fisher Scientific, Waltham, MA, USA) custom-designed for the TMEM260 gene, was performed for all 104 patients with PTA or pulmonary atresia with ventricular septal defect (PA-VSD). The gDNA samples were used to construct amplicon libraries covering the targeted exons and their boundary sequences using the Ion AmpliSeq^TM^ Library Kit 2.0, Ion Xpress^TM^ Barcode Adapters, and Ion OneTouch^TM^ 2 system (Thermo Fisher Scientific) according to the manufacturer's instructions. The constructed libraries were sequenced with an Ion Torrent^TM^ Personal Genome Machine (PGM^TM^) using an Ion PGM^TM^ Hi-Q View Sequencing Kit (Thermo Fisher Scientific), according to the manufacturer's instructions. Raw sequencing data were processed using Torrent Suite^TM^ Software version 5 (Thermo Fisher Scientific) on the hg19 human genome assembly. Functional and allele frequency annotations of detected variants were performed using wANNOVAR[2]. Japanese-specific allele frequency data were obtained using jMorp (<https://jmorp.megabank.tohoku.ac.jp/>)[3]. Variants were filtered as follows: variants with a minor allele frequency (MAF) of less than 3%; variants in the exon or splice region; and variants of indel, nonsynonymous, or nonsense type. The MAF in controls was calculated by analyzing the WES data of controls or by Sanger sequencing with the primers used for the validation of variants in probands.

**Titles and legends to the supplementary figures and tables**

**Figure S1. Flowchart of the case selection process for analysis in the Japanese genome bank for heart disease.**

PA-VSD, pulmonary atresia with ventricle septal defect; PTA, persistent truncus arteriosus.

**Figure S2. *TMEM260* mRNA expression was significantly decreased in the cases with homozygous c.1617del variant**

Relative quantity of *TMEM260* mRNA was examined by reverse transcription-quantitative polymerase chain reaction with *TMEM260* Taqman probe (Hs00956297_m1, Thermo Fisher Scientific) using total RNA extracted from the lymphoblastoid cell line of the cases with homozygous c.1617del variant (n = 4) and inhouse controls (n = 6). Hypoxanthine phosphoribosyl transferase Taqman probe (Hs02800695_m1, Thermo Fisher Scientific) was used as an internal control. **, *p*<0.01 by Welch’s test.

**Figure S3. Pedigree of two families with heterozygous c.1617del variant of *TMEM260* and their sequence chromatograms.**

Participants with and without cardiac defect are shown as filled and unfilled symbols, respectively. Sanger sequencing only for F7-II-2 was performed using the lymphoblastoid cell line. NA, not analyzed; PTA, persistent truncus arteriosus; VT, variant type; WT, Wild type.

**Table S1. Primers used for validation of the *TMEM260* variant and cloning of the *Tmem260* gene.**

VT, variant type; WT, wild type.

**Table S2. Minor allele frequency of *TMEM260*; c.1617del: p.W539Cfs*9 (rs773849415).**

**Table S3. Phenotype information for previously reported probands with *TMEM260* variants.**

ARSCA, aberrant right subclavian artery; ASD, atrial septal defect; Cr, creatinine; IAA, interruption of the aorta; NA, not analyzed; PA-VSD, pulmonary atresia with ventricular septal defect; PTA, persistent truncus arteriosus; RAA, right aortic arch; VSD, ventricular septal defect; -, none.

**Table S4. List of the phenotype information for 2 newly reported patients with heterozygous *TMEM260* c.1617del variant.**

ALSCA, aberrant left subclavian artery; IAA, interruption of the aorta; NA, not analyzed; OFT, outflow tract; PTA, persistent truncus arteriosus; RAA, right aortic arch; -, none.

**References**

1. Wang K, Li M, Hakonarson H. ANNOVAR: functional annotation of genetic variants from high-throughput sequencing data. Nucleic Acids Res. 2010;38:e164.

2. Chang X, Wang K. Wannovar: Annotating genetic variants for personal genomes via the web. J Med Genet. 2012;49:433–6.

3. Tadaka S, Hishinuma E, Komaki S, Motoike IN, Kawashima J, Saigusa D, et al. jMorp updates in 2020: Large enhancement of multi-omics data resources on the general Japanese population. Nucleic Acids Res. 2021;49:D536–44.

4. Hegazi K, Miyake N, Yoshimura J, Okamura K, Niihori T, Saitsu H, et al. Human genetic variation database, a reference database of genetic variations in the Japanese population. J Hum Genet. 2016;61:547–53.

5. Karczewski KJ, Francioli LC, Tiao G, Cummings BB, Alföldi J, Wang Q, et al. The mutational constraint spectrum quantified from variation in 141,456 humans, Genome Aggregation Database Consortium. Nature. 2020;581:434–43.

6. Larsen ISB, Povolo L, Zhou L, Tian W, Mygind KJ, Hintze J, et al. The SHDRA syndrome-associated gene TMEM260 encodes a protein-specific O-mannosyltransferase. Proceedings of the National Academy of Sciences. 2023;120:e2302584120.

7. Pagnamenta AT, Jackson A, Perveen R, Beaman G, Petts G, Gupta A, et al. Biallelic TMEM260 variants cause truncus arteriosus, with or without renal defects. Clin Genet. 2022;101:127–33.

8. Peng M, Jing S, Duan S, Lu G, Zhou K, Hua Y, et al. A novel homozygous variant of TMEM260 induced cardiac malformation and neurodevelopmental abnormality: case report and literature review. Front Med (Lausanne). 2023;10:1157042.

9. Ta-Shma A, Khan TN, Vivante A, Willer JR, Matak P, Jalas C, et al. Mutations in TMEM260 Cause a Pediatric Neurodevelopmental, Cardiac, and Renal Syndrome. Am J Hum Genet. 2017;100:666–75.

10. Kuroda Y, Saito Y, Enomoto Y, Naruto T, Mitsui J, Kurosawa K. PHACES-like syndrome with TMEM260 compound heterozygous variants. Am J Med Genet A. 2023;191:2215–8.
