# Supplementary material for: The c.1617del variant of TMEM260 is identified as the most frequent single gene determinant for Japanese patients with a specific type of congenital heart disease
Source: J Hum Genet. 2024 Feb 26;69(5):215–22. doi: 10.1038/s10038-024-01225-w (PMC11043032; doi:10.1038/s10038-024-01225-w)
Supplement: Supplementary file 2 — Figure S1 [file 10038_2024_1225_MOESM2_ESM.pptx]

## Slide 1
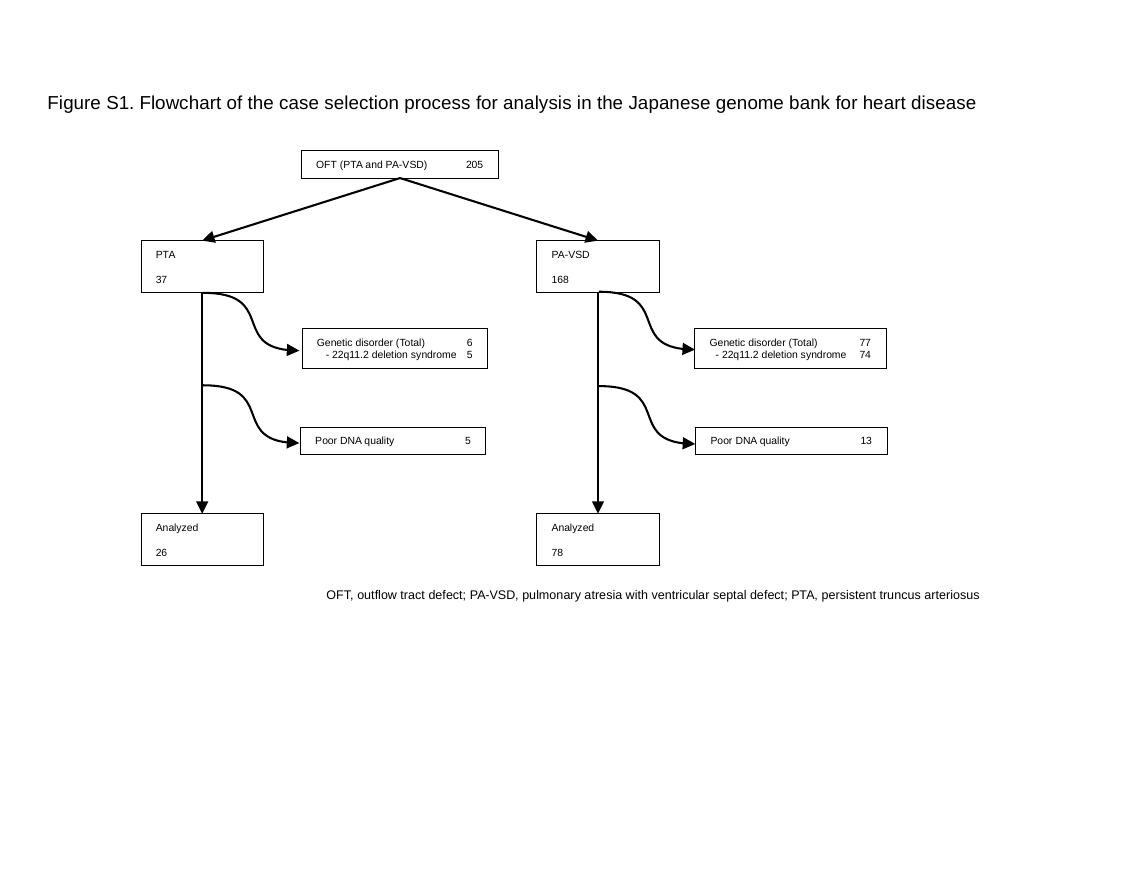

Figure S1. Flowchart of the case selection process for analysis in the Japanese genome bank for heart disease
OFT (PTA and PA-VSD)	205
PTA	37
PA-VSD	168
Genetic disorder (Total)	6
 - 22q11.2 deletion syndrome	5
Genetic disorder (Total)	77
 - 22q11.2 deletion syndrome	74
Poor DNA quality	5
Poor DNA quality	13
Analyzed	26
Analyzed	78
OFT, outflow tract defect; PA-VSD, pulmonary atresia with ventricular septal defect; PTA, persistent truncus arteriosus
