# Supplementary material for: The c.1617del variant of TMEM260 is identified as the most frequent single gene determinant for Japanese patients with a specific type of congenital heart disease
Source: J Hum Genet. 2024 Feb 26;69(5):215–22. doi: 10.1038/s10038-024-01225-w (PMC11043032; doi:10.1038/s10038-024-01225-w)
Supplement: Supplementary file 3 — Figure S2 [file 10038_2024_1225_MOESM3_ESM.pptx]

## Slide 1
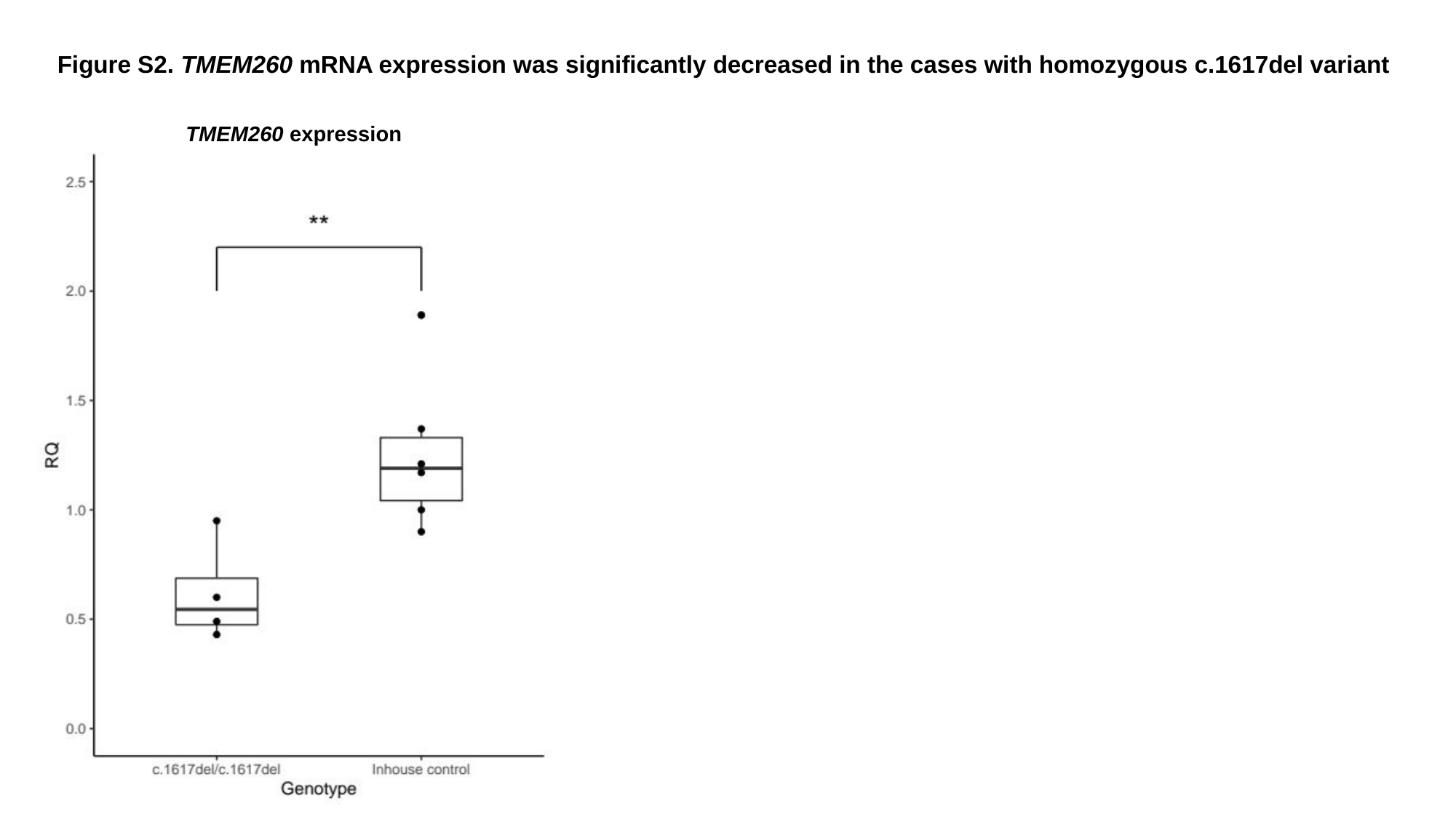

Figure S2. TMEM260 mRNA expression was significantly decreased in the cases with homozygous c.1617del variant
TMEM260 expression
