# Supplementary material for: The c.1617del variant of TMEM260 is identified as the most frequent single gene determinant for Japanese patients with a specific type of congenital heart disease
Source: J Hum Genet. 2024 Feb 26;69(5):215–22. doi: 10.1038/s10038-024-01225-w (PMC11043032; doi:10.1038/s10038-024-01225-w)
Supplement: Supplementary file 4 — Figure S3 [file 10038_2024_1225_MOESM4_ESM.pptx]

## Slide 1
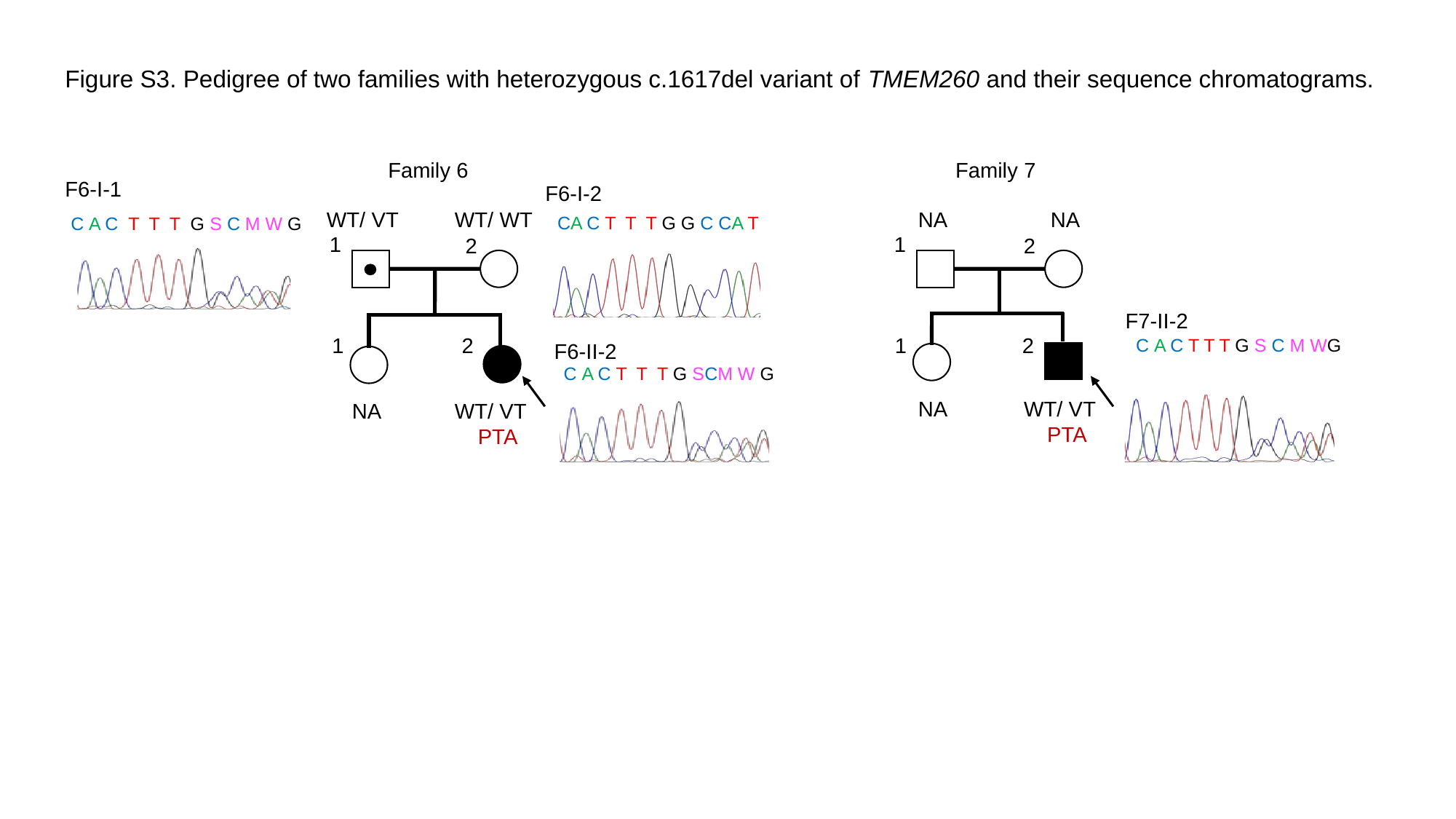

Figure S3. Pedigree of two families with heterozygous c.1617del variant of TMEM260 and their sequence chromatograms.
Family 6
Family 7
F6-I-1
F6-I-2
WT/ VT
WT/ WT
NA
NA
 CA C T T T G G C CA T
 C A C T T T G S C M W G
1
1
2
2
F7-II-2
1
2
1
2
C A C T T T G S C M WG
F6-II-2
 C A C T T T G SCM W G
NA
WT/ VT
PTA
NA
WT/ VT
PTA
