# Supplementary material for: The c.1617del variant of TMEM260 is identified as the most frequent single gene determinant for Japanese patients with a specific type of congenital heart disease
Source: J Hum Genet. 2024 Feb 26;69(5):215–22. doi: 10.1038/s10038-024-01225-w (PMC11043032; doi:10.1038/s10038-024-01225-w)
Supplement: Supplementary file 5 — Table S1 [file 10038_2024_1225_MOESM5_ESM.docx]

**Table S1 Primers used for validation of the *TMEM260* variant and cloning of the *Tmem260* gene.**

| **Name** | **Sequence (5'->3')** | **Target** | **Product size** |
| --- | --- | --- | --- |
| TMEM260_F1 | CTGTTTCCAACAGAAAAGAAACA | c.1617del  Sanger sequence | 392-bp |
| TMEM260_R1 | CCTCTCTCCCCATTGTTCTGTA |  |  |
| Tmem260_F1 | TCCGGGCCCATGGGTCTCCAT | Tmem260 cDNA | 2160-bp |
| Tmem260_R1 | GGAGCAGAGCGGTAAGCAGGGCTAC |  |  |
| Subcloning_SalI | AAGATATCATGGGTCTCCATGGCGACG  SalI | Tmem260  subcloning | WT; 2123-bp  VT; 1781-bp |
| Subcloning_EcoRV | AAGTCGACTCAGACCTTTTTTATATTTCTTAGAC  EcoRV |  |  |

VT, variant type; WT, wild type
