# Supplementary material for: The c.1617del variant of TMEM260 is identified as the most frequent single gene determinant for Japanese patients with a specific type of congenital heart disease
Source: J Hum Genet. 2024 Feb 26;69(5):215–22. doi: 10.1038/s10038-024-01225-w (PMC11043032; doi:10.1038/s10038-024-01225-w)
Supplement: Supplementary file 6 — Table S2 [file 10038_2024_1225_MOESM6_ESM.docx]

Table S2. Minor allele frequency of *TMEM260*; c.1617del: p.W539Cfs*9 (rs773849415)

| Dataset | Population | | Allele Count | Allele number | Allele Frequency | Reference |
| --- | --- | --- | --- | --- | --- | --- |
| In house control | Japanese | | 1 | 476 | 0.0021 |  |
| HGVD | Japanese | | 7 | 2 411 | 0.0029 | [4] |
| jMorp 54K JPN | Japanese | | 392 | 108 604 | 0.0036 | [3] |
| gnomAD | East Asian | Japanese | 0 | 152 | 0 | [5] |
|  |  | Korean | 5 | 3 812 | 0.0013 |  |
|  |  | Other East Asian | 0 | 14 424 | 0 |  |
|  |  | total | 6 | 19 948 | 0.0003 |  |
|  | African/African American | | 0 | 24 934 | 0 |  |
|  | Latino/Admixed American | | 0 | 35 406 | 0 |  |
|  | Ashkenazi Jewish | | 0 | 10 364 | 0 |  |
|  | European (Finnish) | | 0 | 25 096 | 0 |  |
|  | European (non-Finnish) | | 0 | 129 034 | 0 |  |
|  | South Asian | | 0 | 30 612 | 0 |  |
|  | Other | | 0 | 7 216 | 0 |  |
|  | Total | | 6 | 282 610 | 0.000021 |  |
| Allele Frequency  Aggregator | Asian | | 0 | 108 | 0 | * |
|  | South Asian | | 0 | 94 | 0 |  |
|  | African | | 0 | 2 294 | 0 |  |
|  | Latin American 1 | | 0 | 146 | 0 |  |
|  | Latin American 2 | | 0 | 610 | 0 |  |
|  | European | | 0 | 6 962 | 0 |  |
|  | Other | | 0 | 466 | 0 |  |
|  | Total | | 0 | 10 680 | 0 |  |

* Website ([www.ncbi.nlm.nih.gov/snp/docs/gsr/alfa](http://www.ncbi.nlm.nih.gov/snp/docs/gsr/alfa/))
