# Supplementary material for: The c.1617del variant of TMEM260 is identified as the most frequent single gene determinant for Japanese patients with a specific type of congenital heart disease
Source: J Hum Genet. 2024 Feb 26;69(5):215–22. doi: 10.1038/s10038-024-01225-w (PMC11043032; doi:10.1038/s10038-024-01225-w)
Supplement: Supplementary file 8 — Table S4 [file 10038_2024_1225_MOESM8_ESM.docx]

Table S4. List of the phenotype information for 2 newly reported patients with heterozygous *TMEM260* c.1617del variant

| **Family** | **Individual**  **ID** | **Gender** | **Age** | **Race** | **Consanguinity** | **Genotype** | **Cardiac phenotype** | | **Other SHDRA-related phenotype** | |
| --- | --- | --- | --- | --- | --- | --- | --- | --- | --- | --- |
|  |  |  |  |  |  |  | **OFT** | **Aorta** | **Neurological** | **Renal** |
| Family 6 | F6-II-2 | F | 5 years | Japanese | - | hetero | PTA | RAA | - | - |
| Family 7 | F7-II-2 | M | 10 days | Japanese | - | hetero | PTA | IAA  ALSCA | NA | - |

ALSCA, aberrant left subclavian artery; IAA, interruption of the aorta; NA, not analyzed; OFT, outflow tract; PTA, persistent truncus arteriosus; RAA, right aortic arch; -, none.
